# The Early Diagnosis of Bright's Disease

**Published:** 1896-06-27

**Authors:** 


### The Early Diagnosis of Bright's Disease.

Bright's disease is so serious, and, ultimately, so fatal a malady, and yet is one the early stages of which so often remain undiscovered, that, while admitting the difficulties of an early diagnosis, it may be well to lay emphasis on the great importance of taking everv care to avoid the chance of error. When the classical symptoms are fairly pronounced the difficulty vanishes, greatly, sometimes, to the glory of the consultant who may happen to be called in when all has become plain, and greatly to the discomfiture of the practitioner who, having failed to form a correct diagnosis at a period when, in fact, diagnosis was difficult, if not impossible, has drifted on and missed the meaning of the signs which have one by one developed, while, as in a dissolving view, the picture of the casual ailment has been gradually turning into that of the serious disease.

It is a cheap and easy triumph for the consultant, but is none the less injurious to the reputation of the practitioner, and we can but urge the great importance, during the treatment of any chronic ailment, not only of going over the whole case thoroughly at first, but of repeating this formal investigation periodically. Too often the patient's manner of dealing with his doctor throws difficulties in the way of completely investigating his disorder. He goes to a consultant in all seriousness, probably by appointment, and with plenty of time on hand. But in visiting his own doctor he "just looks in," and asks for a bottle or something for "my old indigestion," or for "my confounded headache again," as the case may be; and this habit of mentally ticketing his ailment and asking for relief for one prominent symptom has in the end an influence on the doctor also. The mental ticket remains unchanged; and it is to be feared that many a good man who, if he had but begun at the beginning and gone through the whole case, would have diagnosed its nature as certainly as the most celebrated of consultants, misses the point by yielding to the position forced on him by the patient and prescribing merely for what is most complained of.

Perhaps, also, he sometimes hesitates to incur the odium of being thought to be "making a job for himself," in the elegant phraseology of the public, by treating seriously the "bit of indigestion" or the "touch of asthma" of which his patient complains.

At any rate, it is a fact that in the post-mortem room the kidneys are often found to be affected with interstitial disease in patients who have died of other ailments without any attention having ever been attracted to these organs. The onset of this disease is marked by but few distinctive features, and, indeed, the symptoms which occur may, almost any of them, appear devoid of deeper meaning if they be taken individually. It is their grouping which is so significant of danger, and makes it so plain that they have some hidden malady as an underlying cause. The recognition of the real meaning of apparently isolated symptoms, the mere fringe of an unsuspected malady, is a far better test of the acumen and sagacity of the practitioner than the diagnosis of the typical disease.

In middle-aged people recurring headaches are often put down to over work, want of appetite, to confinement in close, gas-lighted offices, and nausea or vomiting to the alcohol which the patient may be known to be taking in more than necessary quantity; yet each of these symptoms is but too often a mere sign of kidney disease, and in fact the growing love of alcohol may itself only have occurred in response to the growing weakness caused by that malady.

An attack of "asthma" in the night in a man not used to such attacks may be truly asthmatic, or, being relieved by carminatives, may be put down to stomach. But then it may be but the first warning of failure in the hypertrophied heart of Bright's disease.

The same in regard to palpitation, shortness of breath, certain affections of sight, bleeding from the nose, and the crowd of nervous symptoms covered by the word neurasthenia. Taken together there is no difficulty, but coming singly it is easy to be misled unless one is constantly suspicious and continually on the look out.

Diagnostic skill lies more in suspecting always that things are not so simple as they seem, and in the habit of going right through the case, than in any very extraordinary genius.

